# Determining the Permeability of Porous Bioceramic Scaffolds: Significance, Overview of Current Methods and Challenges Ahead

**DOI:** 10.3390/ma17225522

**Published:** 2024-11-12

**Authors:** Roberta Gabrieli, Alessandro Schiavi, Francesco Baino

**Affiliations:** 1 Institute of Materials Physics and Engineering, Department of Applied Science and Technology, Politecnico di Torino, Corso Duca Degli Abruzzi 24, 10129 Turin, Italy; roberta.gabrieli@polito.it; 2 National Institute of Metrological Research (INRiM), Applied Metrology and Engineering Division, 10135 Turin, Italy; a.schiavi@inrim.it

**Keywords:** bioactive glass, hydroxyapatite, porosity, fluid flow, bone tissue engineering

## Abstract

The “architectural suitability” of scaffolds for bone tissue engineering is commonly evaluated by assessing the pore volume and the mean pore size (or pore size distribution, if possible) and comparing these values with the reference ranges of human cancellous bone. However, these two parameters cannot precisely describe the complex architecture of bone scaffolds and just provide a preliminary comparative criterion. Permeability is suggested as a more comprehensive and significant parameter to characterize scaffold architecture and mass transport capability, being also related to bone in-growth and, thus, functional properties. However, assessing the permeability of bioactive ceramics and glass scaffolds is a complex task from both methodological and experimental viewpoints. After providing an overview of the fundamentals about porosity in scaffolds, this review explores the different experimental and numerical approaches used to determine the permeability of porous bioceramics, describing the methodologies used (pump-based, gravity-based, acoustic and computational methods) and highlighting advantages and limitations to overcome (e.g., reliability issues and need for better standardization of the experimental procedures).

## 1. Introduction

The loss of tissue, both in terms of structure and function, is a problem that involves great costs for healthcare worldwide [1]. Driven by the increasing demand for therapies to address tissue defects caused by injury, trauma, disease or age-related degeneration, tissue engineering holds a tremendous potential for revolutionizing healthcare [2,3].

Tissue engineering represents an interdisciplinary discipline that combines principles of biomaterials, engineering, biology and medicine to design and develop innovative solutions for the regeneration and repair of biological tissues that have been damaged or lost [1,4]. This field relies on the use of a wide range of biocompatible materials, primary or typically stem cells, growth factors and tissue engineering techniques to produce constructs that mimic the complexity and functionality of native biological tissues.

If we consider that bone is the second tissue of the body requiring repair or regeneration after blood [5], it is not surprising that a lot of biomedical research is addressed to biomaterials for bone tissue engineering [6]. Bone regeneration can be achieved by using transplant tissues (e.g., autografts) or man-made implantable materials, such as bioceramics [7]. Some of them, including calcium phosphates [8] and bioactive glasses [9], are routinely used in orthopedics and dentistry with very good clinical outcomes.

In tissue engineering, biomaterials are often designed and produced as porous structures called “scaffolds”, which aim to create a favorable environment resembling the natural conditions of the body, facilitating cell growth, differentiation and proliferation. Indeed, the microstructure of the scaffold and the related biological performances are very crucial to maintain, support and even stimulate the specific functions of the bone tissue.

The three-dimensional (3D) architecture of the scaffold includes several key elements (e.g., pore size and shape, open porosity, surface topography) that influence vascularization and fluid flow through the material. For instance, interconnected pores allow the passage of nutrients and waste products, the distribution of channels or pores facilitates the flow of fluids and the shape and geometry of internal pores are also other structural properties affecting osteoconductivity and mechanical properties [10]. Overall, the physical and chemical surface properties of the scaffold are key to dictating biological responses, including the attachment and proliferation of cells [1,11]. Previous studies have found that cell growth within a scaffold is highly dependent on the effectiveness of nutrient permeation through the porous structure [12,13].

Overall, the microstructural parameters that characterize the scaffold, such as pore interconnectivity and tortuosity, are closely related to the mass transport phenomena within the porous network and estimating them accurately is indeed a complex task [14]. Permeability is a crucial factor because it determines the ease with which nutrients, waste products and biomolecules can move through the scaffold, directly impacting cell survival, growth and tissue regeneration. Therefore, intrinsic permeability is recognized as a key parameter for describing the scaffold architecture related to the physical properties of mass transport [13,15]. Permeability quantifies the ability of a scaffold to allow the passage of fluids through its porous structure, where higher values of permeability enhance bone regeneration [15].

Given the pivotal role of permeability on the performance of bone tissue engineering scaffolds, in this work, we discuss the main methodologies and approaches to assess this property in porous bioceramics. The literature search was performed in the database SCOPUS using the keywords “bioceramics”, “bioactive glass”, “scaffold”, “permeability” and their combinations. The first part of this review illustrates the parameters that influence permeability in bone tissue engineering scaffolds, and then the most popular and innovative experimental methods and computational approaches to estimate it are discussed.

## 2. Parameters That Influence Permeability

The effectiveness of a scaffold in supporting tissue growth and regeneration depends largely on how well it facilitates the mass (or fluid) transport processes [13,16]. Permeability is a critical feature that influences the scaffold biological performance and, in turn, depends on several parameters such as porosity, pore size, pore interconnectivity, tortuosity and surface area, as summarized in [17,18].

Porosity refers to the proportion of the scaffold volume that consists of empty spaces. Total porosity (*P_tot_*) is measured as:

(1) $$P_{tot}=1-\frac{\rho_{scaffold}}{\rho_{material}}\;\;$$

where *ρ_material_* is the density of the non-porous material of which the scaffold is fabricated and *ρ_scaffold_* is the density of the scaffold measured by a gravimetric method, dividing its mass by its volume. Unlike total porosity, effective porosity, ε, only accounts for the interconnected pores that allow fluid to move through the scaffold, without considering close pores and dead-end pores. This makes it a more reliable indicator of permeability.

Average pore size and pore size distribution are other microstructural features that influence permeability. Based on the literature [19], the minimum recommended pore size for a scaffold is 100 µm [20], but other studies have shown better osteogenesis for implants with pores greater that 300 µm in order to allow fluid flow more easily [21]. Pores well distributed throughout the scaffold volume typically involve the uniform distribution of nutrients and oxygen, which are critical for cell growth and tissue development. As with pore distribution, the interconnectivity of pores is also crucial for nutrient permeability and the effective transport of fluids throughout the scaffold. However, there is an upper limit for porosity and pore size to consider due to mechanical property constraints; increasing the void volume reduces the mechanical strength of the scaffold, which is fundamental to promote bone regeneration. Therefore, bioceramic scaffolds having more than 85 vol.% of pores are typically too mechanically weak to figure out any practical use in the clinic [22].

Pore sphericity, which is a quantification of pore shape, refers to how closely the shape of the pores resembles a sphere. Pores with higher sphericity generally provide a more uniform pathway for fluid to flow; conversely, irregularly shaped pores may create more resistance to the fluids that move through the scaffold [23]. Another feature that may prevent fluid from passing through is the narrowing of the pores; the fluid movement becomes more restricted, increasing resistance to flow and thereby reducing permeability [24,25]. Being linked to these aspects, tortuosity plays a crucial role in the fluid flow [26,27]. Tortuosity is expressed as:

(2) $$\tau =\frac{L_{p}}{L_{s}}\;\;$$

where *L_p_* refers to the effective pore length and *L_s_* refers to the sample length. This term is linked to the complexity of the fluid pathways within the scaffold. Higher tortuosity indicates more complicated pathways, which increases resistance to fluid flow. Furthermore, tortuosity was reported to play a role in mechanical properties, as the mechanical strength of trabecular structures increases as tortuosity decreases [28].

The scaffold surface area, or the total area available for fluid interaction, influences how easily fluids can pass through and it is the area available for cell adhesion. Improving these parameters is key to creating scaffolds that successfully support tissue growth by making sure nutrients and waste are properly transported.

It is worth mentioning that, apart from the microstructure, other parameters also play an important role in the tissue vascularization of the scaffolds, including the viscosity and the density of the fluid, and biological factors such as cell seeding density and nutrient concentration [14].

Intrinsic permeability can be calculated using Darcy’s empirical law [29,30], which describes flow through a porous medium:

(3) $$\frac{∆P}{L}=\frac{Q_{v}}{k}\cdot \frac{\mu }{A}=\frac{\mu }{k}\cdot U$$

where ∆*P* is the differential pressure across the scaffold, *L* is the thickness of the scaffold, *Q_v_* is the volumetric flow rate, *k* is the intrinsic permeability, *A* is the effective surface area of the sample, *U* is linear flow velocity and *μ* is the fluid dynamic viscosity. Darcy’s law relates the intrinsic permeability to the volumetric flow rate and forced pressure difference in a flow channel when the Reynolds number is lower than 10. This formula relies on several key assumptions to simplify the model. First, it assumes a parabolic velocity profile within the unit cells. Due to the complexity of porous microstructures, the fluid is assumed to flow in a single direction, allowing the pressure gradient to be expressed accordingly; also, the focus is on the smallest volumes that can represent the entire microstructure. Additionally, the fluid flow is assumed to be creeping, incompressible and in a steady state—conditions that are necessary to determine the velocity profile and permeability. If the fluid were not in a steady state (Reynolds number greater than 10), inertial losses would occur, making Darcy’s law inaccurate and requiring a non-Darcian term.

A general equation formulated by Ergun [31] states that the pressure loss is due to the combination of frictional and inertial losses:

(4) $$\frac{∆P}{L}=150\frac{{\left(1-\varepsilon \right)}^{2}}{{D^{2}\varepsilon }^{3}}\cdot \;\mu U+1.75\;\frac{\left(1-\varepsilon \right)}{{D\varepsilon }^{3}}\cdot \rho U^{2}$$

where *ε* is the effective porosity (i.e., only the open porosity allowing the fluid flow through the porous media) and *D* is the pore average diameter; the coefficients 150 and 1.75 are extrapolated by fitting experimental data and could have different values depending on the material topology [32,33].

The first term of Equation (4) is known as the Blake–Kozeny equation and represents the viscous losses in laminar flow; it is equal to Darcy’s law when inertial losses are negligible, i.e., if Re < 10. The second term describes the inertial losses or the kinetic energy loss in a turbulent flow and is called the Burke–Plummer equation [32,33].

Over the years, Equation (4) has been modified by other authors [34,35,36,37]. Namely, it was found that the empirical coefficients 150 and 1.75 can actually be expressed as a function of pore morphology and geometry, with a clear physical meaning, as follows:

(5) $$\frac{∆P}{L}=72\;\tau \frac{{\left(1-\varepsilon \right)}^{2}}{D^{2}\varphi^{2}\;\varepsilon^{3}}\;\cdot \;\mu U+0.75\;\tau \;\frac{\left(1-\varepsilon \right)}{\;D\varphi \;\varepsilon^{3}}\;\cdot \left(\frac{3}{2}+\frac{1}{\beta^{4}}-\frac{5}{2\beta^{2}}\right)\cdot \;\rho U^{2}\;\;\;$$

This general model allows evaluating the resistance of flow through a porous media and takes into account the pore tortuosity *τ*, the pore sphericity *φ*, the pore narrowing ratio *β*, the effective porosity *ε* and the fluid properties.

The pore tortuosity *τ* is the actual (average) length of the pores with respect to the thickness of the porous media in the flow direction, and it can be calculated as a function of effective porosity only, on the basis of several empirical or semi-empirical formulations [38], for example, as follows:

(6) τ=1−0.4ln⁡ε         

**Figure 1:**
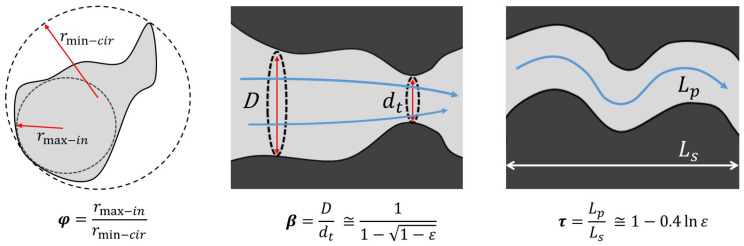
Explanatory image for the calculation of the parameters *φ* (pore sphericity), *β* (pore narrowing ratio) and τ (pore tortuosity) [39].

The pore sphericity *φ* represents how closely the shape of a pore section tends to a perfect circumference (*φ →* 1), or to a flat slit (*φ →* 0), and can be calculated as the ratio between the maximum radius of a circumference inscribed in the pore and the minimum radius of the circumference circumscribing the pore, as displayed in Figure 1. The pore narrowing ratio *β*, being the ratio of *D* to throat diameter *d_t_* [35], is also illustrated in the same figure. Specifically, *β* can also be determined as a function of *ε*, as follows:

(7) $$\beta =\frac{1}{1-\sqrt{1-\varepsilon }}$$

The equivalent pore diameter *D* is calculated as expressed in Equation (8) and is a function of *V_p_*, the volume of a single non-spherical pore and *A_p_*, the surface area of the pore [22]:

(8) $$D=\frac{6\;V_{p}}{A_{p}\;\varphi \;}$$

Considering Equation (3) and assuming a Reynolds number less than 10, the inertial losses depending on *U*^2^ become negligible, leading to the conclusion that the intrinsic permeability in a porous material can be explicitly expressed as a function of pore morphology and geometry, as described in [25,30]:

(9) $$k\;≅\frac{\varepsilon^{3}\;D^{2}\varphi^{2}}{\;72\;\tau {\;\left(1-\varepsilon \right)}^{2}}$$

## 3. Experimental Methods to Evaluate Permeability

The experimental approaches for estimating scaffold permeability can be categorized into direct and indirect methods. Direct methods include all the procedures in which the permeability is measured by a fluid flow through the scaffold, while indirect methods measure permeability from structural and pore characteristics of the scaffold. Examples of indirect methods are mercury intrusion porosimetry, micro-tomographic imaging, physical gas absorption and capillary flow porosimetry. All these methods provide information about the microstructure of scaffolds, which affects fluid transport properties and is related to the overall performance of the scaffold, including permeability. Indirect methods have some limitations due to their assumptions about the scaffold structure and properties, which do not fully describe the overall complexity of the porous material. Among these characterization methods, micro-computed tomography is the most versatile and powerful technique for the non-invasive non-destructive 3D imaging and analysis of materials in medicine, industry and biology. It is a 3D radiographic imaging technique that is conceptually similar to medical computed tomography systems used for clinical diagnosis in hospitals. Unlike such devices, which typically have a spatial resolution of the order of one millimeter, micro-tomography can achieve a submicronic resolution [40]. Microstructural parameters that can be obtained through this method include total, open and closed porosity, pore sphericity, pore interconnectivity and pore size distribution, along with a 3D reconstruction of the entire structure of the porous scaffold. This investigation technique provides a more complete set of information compared, for example, with scanning electron microscopy, which refers to 2D images only.

Direct methods allow measuring the pressure drop and volumetric flow rate through the scaffold and, then, quantifying the permeability using Darcy’s law (Equation (3)) and the Ergun model (Equation (4)). In detail, direct permeability measurement systems include three different techniques: pump-based methods (involving a fluid motion generated by a pump through a scaffold sample), an acoustic method and a gravity-based method (relying on fluid flow driven by gravitational force).

### 3.1. Pump-Based Methods

**Figure 2:**
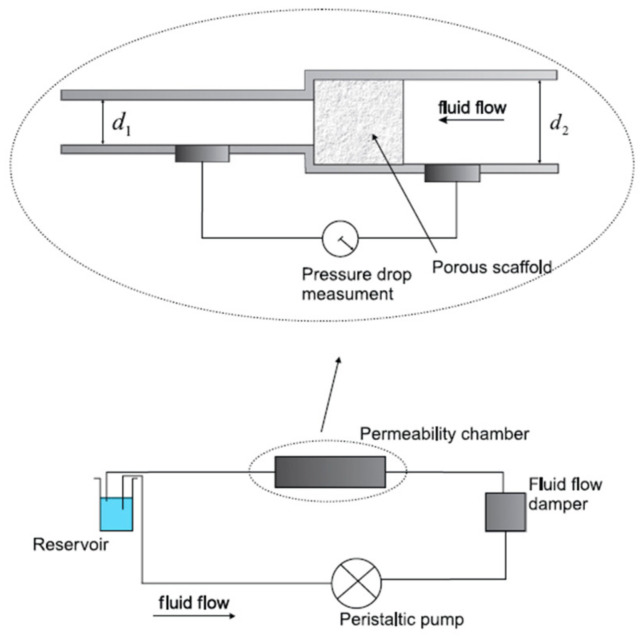
Experimental setup for permeability evaluation through a pump-based method (reproduced from [41]).

The pump-based method for evaluating permeability involves generating fluid motion through a scaffold sample using a pump. The test setup typically includes a permeability chamber or permeameter, a peristaltic pump, a fluid flow damper to eliminate the peristaltic pulses from the pump and ensure a continuous flow rate and a reservoir. In this process, the peristaltic pump moves the fluid, creating a controlled flow through the scaffold. The scaffold, acting as a resistance to flow, causes a pressure drop, which is measured in the permeability chamber where the scaffold is housed [41]. Figure 2 shows a schematic representation of the test bench.

This specific setup was utilized by Ochoa et al. [41] for assessing the permeability of 45S5 Bioglass^®^-based scaffolds to be used for bone tissue engineering applications. The porous sample was placed in the permeability chamber where the pressure drop was measured. The test was performed using deionized water that was taken from a reservoir and was moved by using a peristaltic pump. In this configuration, it was possible to apply Darcy’s law (Equation (3)). The pressure drop related to the scaffold was obtained by subtracting the pressure drop measured in the chamber without the scaffold from the value measured across the chamber with the scaffold inside. Hence, different fluid flow regimes were applied to obtain pressure drop–flow rate curves and the Darcian permeability was eventually estimated.

Recently, Gabetti et al. [42] developed a versatile permeability testing apparatus in compliance with ASTM International F2952-22 standards [43]. This apparatus ensures reliable and repeatable measurements of the intrinsic permeability of porous scaffolds to be potentially used in bone tissue engineering applications. It employs a pump-driven approach and consists of a modular permeability chamber integrated into a closed-loop hydraulic system. This system incorporates a peristaltic pump, pressure sensors and recirculating demineralized water. This permeameter is employed for the determination of permeability of rigid porous scaffolds, such as calcium carbonate bone-mimicking scaffolds, and yields consistent results. In this paper, a detailed uncertainty budget, integrating the experimental evidence, is also provided, along with a comparison with the acoustic method, which is described in Section 3.3.

**Figure 3:**
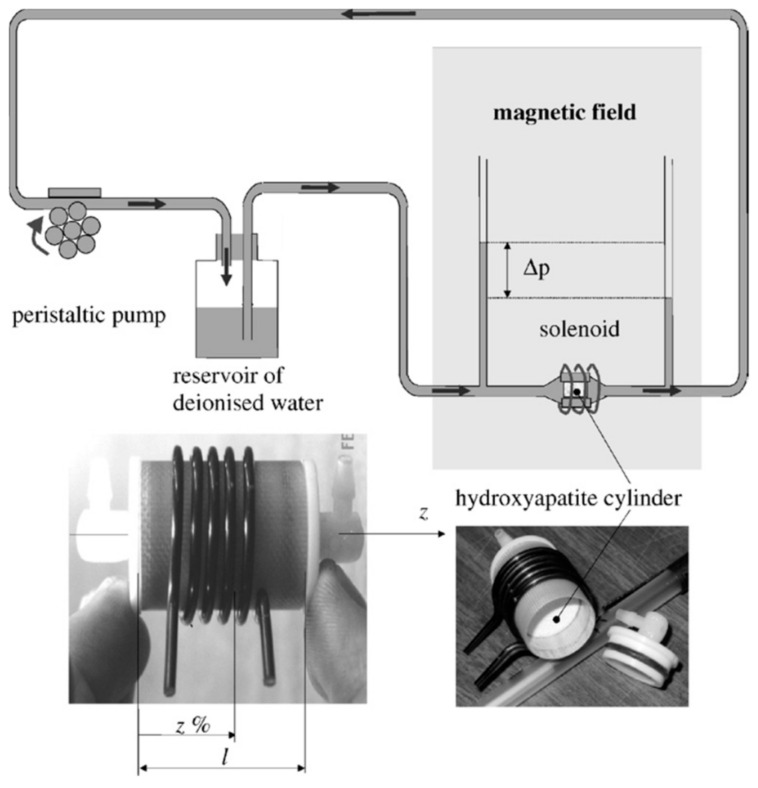
Experimental setup for permeability evaluation through high-resolution MRI (reproduced from [44]).

Swider et al. [44] developed a high-resolution magnetic resonance imaging (MRI) technique to non-destructively characterize permeability, fluid velocity field and shear field within coralline hydroxyapatite implants (Pro Osteon HA500 from Interpore, Irvine, CA, USA) with interconnected porosity. An illustration of the experimental setup is shown in Figure 3.

In this test, the fluid used was deionized water and the flow circuit was characterized by a peristaltic pump and a pressure reservoir to create a constant fluid flow. There were two capillaries used to measure the pressure gradient ∆*p*, determined by the difference between upstream and downstream pressures of the flow chamber and a vacuum was applied to ensure full water saturation of the porous bioceramic implant. The scaffold was closed in an MRI probe consisting of a flow chamber surrounded by a solenoid radio frequency coil and positioned in the MRI scanner in order to evaluate the axial fluid velocity (along the *z*-axis) through the scaffold. For further analysis, each MR acquisition imaging plane was set at *z%* equal to 25%, 50% and 70% of the sample length *l*, as shown in Figure 3. The permeability was calculated from Darcy’s law (Equation (3)), as also reported by Ochoa et al. [41].

**Figure 4:**
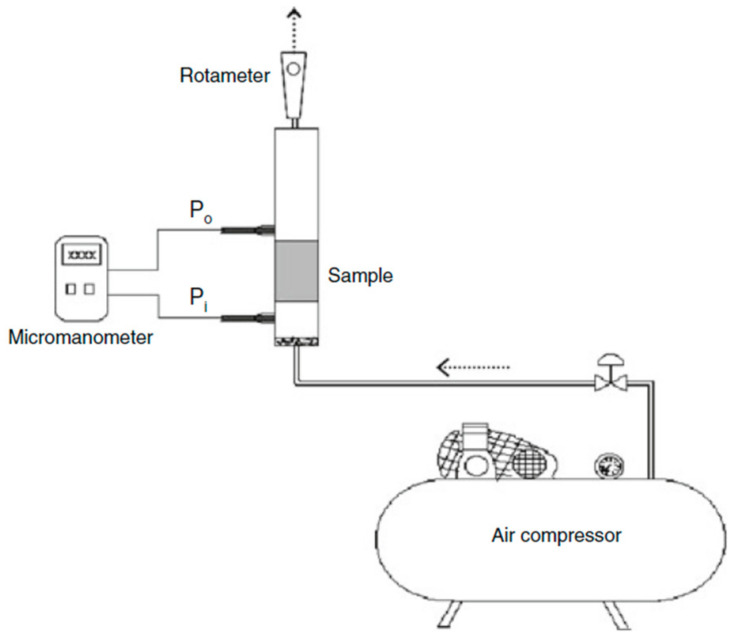
Experimental setup for permeability evaluation using air as flowing medium (reproduced from [45]).

As an alternative to water, Innocentini et al. [45] used air as a fluid to evaluate the permeability of porous hydroxyapatite scaffolds. The air at room temperature was induced to flow through the scaffold at a stationary regime. Figure 4 displays the experimental setup, where the scaffold is sealed between two chambers, and the pressure drop and the volumetric flow rate are measured by a digital micromanometer and a rotameter, respectively.

In this work, the permeability evaluation was based on Forchheimer’s equation:

(10) $$\frac{∆P}{L}=\frac{\mu }{k_{1}}\cdot v_{s}+\frac{\rho }{k_{2}}\cdot v_{s}^{2}\;\;\;$$

The relationship between pressure drop and flow rate derived from the collected data was fitted with a quadratic law; the face velocity vs. the volumetric flow rate *Q* divided by the nominal face area of the sample exposed to flow. With respect to Darcy’s law, the terms *k_1_* and *k_2_* are thus known as Darcian and non-Darcian permeability constants; specifically, the first term in Equation (10) refers to the viscous losses due to the friction between fluid layers, while the second term represents the kinetic energy losses due to the changes in the direction of fluid motion [45].

### 3.2. Gravity-Based Method

**Figure 5:**
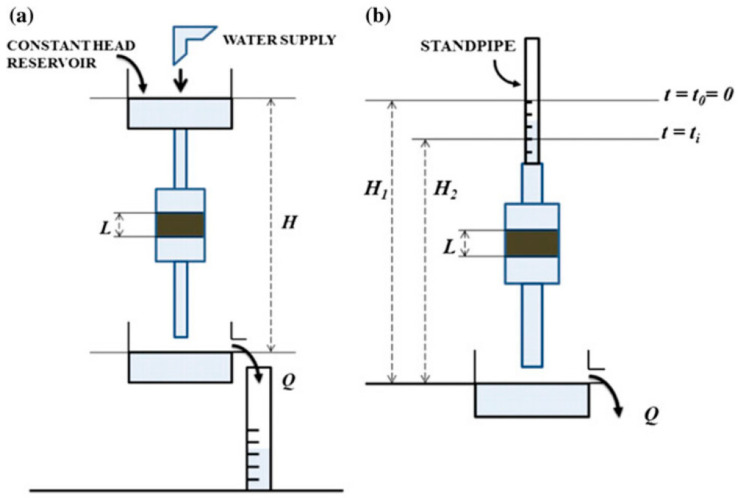
Representation of (**a**) constant head method and (**b**) falling head method (reproduced from [33]).

Gravity-based methods involve all the techniques used to measure the permeability of a material by exploiting the force of gravity to make a fluid flow through the material itself. In this method, the pressure that pushes fluid through the sample is generated solely by the difference in height (or head) of the fluid relative to the sample, without the use of pumps or other mechanical devices. The gravity-based methods can be applied in two different ways, i.e., using either a constant head or a falling head approach [46,47,48], as shown in Figure 5.

In the constant head method, fluid is allowed to flow through the scaffold under a constant pressure head, meaning that the height of the fluid reservoir remains unchanged throughout the experiment. The fluid flow rate is measured while maintaining this steady pressure. The most commonly used fluid is distilled water at a constant temperature. Before starting the test, the scaffold is saturated to remove air bubbles inside the scaffold. The volumetric flow rate is measured by timing the volume of fluid that flows through the scaffold.

The falling head method involves a variable pressure head, where the fluid level in the reservoir decreases over time as it flows through the scaffold. The time taken for the fluid level to drop between two specific points is recorded, and the permeability is calculated based on this change in fluid height and the initial and final pressure heads [33].

In both approaches, the intrinsic permeability is calculated using Darcy’s law equation, expressed as:

(11) $$v=K\cdot \;\frac{H}{L}$$

where *v* is the fluid velocity, *K* is the coefficient of proportionality called hydraulic conductivity, *H* is the distance between two free water surfaces and *L* is the thickness of the scaffold. The hydraulic conductivity *K* of a porous medium is a scalar quantity that measures how easily a fluid is transported through a tortuous void space. The intrinsic permeability *k* is linked to the hydraulic conductivity *K* [49]:

(12) $$k=K\cdot \;\frac{\mu }{\rho g}$$

In the constant head method, the hydraulic conductivity is equal to:

(13) $$K=\frac{Q\;L}{A\;H}\;\;\;$$

where *H* is the constant pressure difference and *Q* is the volumetric flow rate.

In the falling head method, the hydraulic conductivity is equal to:

(14) $$K=\frac{a\;L}{A\;t}\;\;ln\left(\;\frac{H_{1}}{H_{2}}\;\right)\;\;\;$$

where *a* is the cross-sectional area of the standpipe, *H*_1_ and *H*_2_ are the initial and final heights of the fluid and *t* is the time taken for the fluid to drop from height *H*_1_ and *H*_2_.

An example of permeability evaluation using the constant head method is reported by Li et al. [13], where demineralized water was selected as the fluid and the permeability was evaluated for biphasic calcium phosphate and hydroxyapatite macroporous scaffolds.

There are some important differences between pump-based and gravity-based approaches. Pump-based methods generally rely on the measurements of a continuous airflow or waterflow pressure drop, i.e., the differential between upstream and downstream pressures across the scaffold, and the fluid flow velocity through the scaffold. Experimental data are extracted by using several sensors, such as pressure gauges, manometers and flowrate meters, based on different technologies and methods of observation. Gravity-based methods typically involve the measurement of height differences along capillaries and the increasing weight of liquid in a reservoir over time by using optical techniques and precision balances.

Although these two methods are apparently intuitive and simple, the occurring uncertainties and measurement errors are very difficult to accurately identify. In fact, in-depth analyses of the possible causes of measurement errors and the impact of their propagation in experimental data are not provided. The lack of this fundamental information does not allow identifying the accuracy and precision of the experimental results obtained by using the systems described above (except for the method described in [42]).

### 3.3. Acoustic Method

An acoustic approach to evaluate the intrinsic permeability of bioceramic scaffolds was originally proposed and applied to commercial calcium carbonate porous implants by Schiavi et al. [32]; then, the method was progressively refined and successfully extended to hydroxyapatite [50] and bioactive glass scaffolds [39] with foam-like architectures. The test bench provides a rapid permeability measurement, based on the accurate quantification of the acoustic pressure wave drop of an alternating airflow through the samples, which is generated by an oscillating piston in a closed cavity of known geometry. The scaffold is located in a proper seat of the wall of the measurement chamber; the pressure wave drop in the closed cavity is detected by using a single low-frequency pressure field microphone. The sensitivity of the acoustic permeameter is established from a direct microphone calibration (in mV/Pa_cal_) by using the system as a pistonphone. By generating a slow alternating airflow through the scaffold and measuring the pressure differential, this acoustic method calculates the permeability based on Darcy’s law for oscillating flows according to the following experimental model [32]:

(15) $$k_{D}=\mu \frac{q_{v,rms}}{p_{rms}}\cdot \frac{L_{s}}{A_{s}}\cdot \zeta =\mu \frac{\omega \partial V{\left(\sqrt{2}\right)}^{-1}}{\gamma p_{0}\partial V{\left(V_{0}\sqrt{2}\right)}^{-1}}\cdot \frac{L_{s}}{A_{s}}\cdot \zeta =\mu \frac{\omega V_{0}}{\gamma p_{0}}\cdot \frac{L_{s}}{A_{s}}\cdot \zeta \;\;\;$$

where *q_v,rms_* is the alternating r.m.s. volumetric airflow *(q_v_*_,rms_ *= U∙A_s_*), *p*_rms_ is the sinusoidal r.m.s. pressure component depending on the atmospheric static pressure *p*_0_ (with heat capacity ratio γ = 1.4), *∂V* is the volume variation induced by the motion of the piston on the volume of air *V*_0_, *L_s_* is the length of the porous medium (along the flow direction), *A_s_* is the cross-sectional area of the porous medium perpendicular to the flow direction, *ω* is the frequency of the airflow oscillation and *ζ* is the experimental ratio between the dynamic pressure waves as assessed from microphone indications (in mV/Pa_meas_), with respect to calibration (in mV/Pa_cal_).

**Figure 6:**
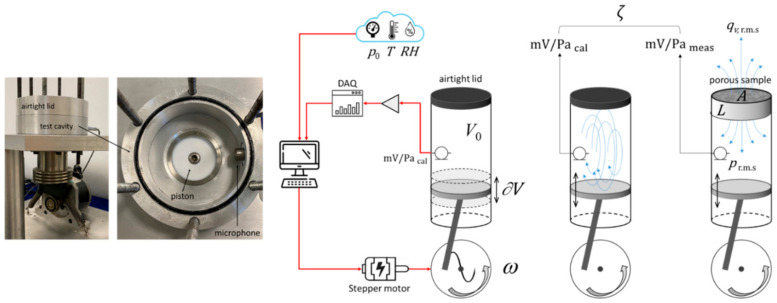
Experimental setup for the acoustic permeameter and scheme of the technical method, along with the related experimental quantities.

This approach simplifies the measurement process and is particularly effective for highly porous scaffolds, as illustrated in Figure 6.

The acoustic permeameter allows performing very accurate measurements since its sensitivity is established from a direct calibration of the microphone inside the cavity, as schematically shown in Figure 6. A detailed uncertainty budget of the measurements performed by using the acoustic permeameter, according to the GUM rules [51], and the occurring errors propagation were analyzed and comprehensively discussed in [32,50] and [39]. Moreover, a rigorous bilateral proficiency test was recently carried out by comparing blind data measured by the acoustic permeameter and by the pump-based method according to the procedure described in the ASTM International F2952-22 standard, providing highly compatible results, especially in terms of repeatability and reproducibility [42].

## 4. Computational Methods to Evaluate Permeability

In recent decades, advances in computer performance have enabled direct simulation of complex fluid behavior inside scaffolds. Computational fluid dynamics (CFD) is used to solve Navier–Stokes equations numerically and Darcy’s law at the microscopic level, and then upscaled using homogenization methods to obtain macroscopic information. Combining the reconstruction of realistic 3D models of porous scaffolds from imaging with CFD provides insights not only into fluid transport through the scaffold but also into the distribution of friction forces inside the 3D matrix, which are crucial in mechanobiology and are difficult, if not impossible, to measure experimentally [33,52]. Several computational methods, including the finite element method (FEM), finite volume method (FVM), finite difference method (FDM) and lattice Boltzmann method (LBM), are well suited for simulating flow through scaffolds with both regular and irregular geometries, enabling the correlation of permeability with porosity, specific surface area, tortuosity and shear stress.

FDM uses grids for the discretization of the scaffold geometry where it is possible to apply the governing equation but it is inefficient for complex geometries [53].

FEM is a numerical tool used to approximate solutions to complex partial differential equations (PDEs) in real-time engineering problems [54]. FEM requires division of the scaffold geometry into smaller regions, called elements, through the process of discretization. Tetrahedral elements with nodes in the vertexes are commonly used for the discretization of complex geometries. The collection of these interconnected elements and nodes forms the finite element mesh, which is essential for simulating low-velocity flow where Darcy’s law can be applied to evaluate the permeability. Increasing the number of finite elements improves the resolution and the accuracy of the simulation but results in an increase of computation time [54,55]. Comparatively, FVM relies on dividing the geometry into more controlled volumes centered around mesh points and provides the facility to solve complex numerical models also for complex curved geometries [56]. This method is considered more efficient because it focuses directly on the conservation of physical quantities across each volume and provides more accurate results with reduced computational time [55].

Voronov et al. [57] employed the lattice Boltzmann approach to calculate the permeability of scaffolds with different geometries. LBM is used to simulate the fluid flow within porous scaffolds, particularly for irregular scaffold geometries by solving the discrete Boltzmann equation. The Boltzmann equation refers to the particle distribution function, which is a statistical model describing how particle distribution changes as a function of time and space. LBM approximates the fluid flow by simulating the movement and collisions of particles across a grid. Once particles have moved and collided, LBM calculates macroscopic fluid properties (e.g., density and velocity) by summing the properties of particles in each direction at every grid node. This allows to map fluid behavior throughout the scaffold.

In some studies [52,58], the effective permeability of scaffolds is evaluated using a mathematical approach based on Darcy’s law and homogenization methods that allow understanding not only the permeability values associated to a specific design but also the relationship between permeability and effective porosity, pore size and scaffold geometry.

Probably the first computational study addressed for estimating the permeability of bioceramic scaffolds was reported by Jones et al. [59], who used the 3D reconstruction of bioactive glass foam geometry obtained via micro-tomographic analysis in a microscale flow simulation. The permeability was calculated by numerically solving the Navier–Stokes equations, which describe the flow within the porous scaffold at the local scale through a code originally developed for water flowing between rocks.

In the study by Dias et al. [52], the scaffolds were simplified to have a periodic microstructure, where the scaffold was made by a repetition of a unit cell. In this case, the homogenization method allows avoiding the complex analysis of the second order differential equation for the evaluation of the permeability. Sanz-Herrera et al. [58] calculated the permeability values by homogenization theory for some representative volume elements of the microstructure of the doped dioxide zirconium scaffolds.

Results obtained in these two studies compared the permeability values obtained from the experimental test with the numerical results and they are consistent also with other results in the literature. The computational approach is not only a method for evaluating the permeability while avoiding time-consuming experiments that may need expensive and custom-made tools but it may also become an attractive tool for scaffold design.

An analytical fractal-based model to calculate the permeability in porous media was first proposed by Yu and Li in 2001, and then refined by Yu in 2005 [60,61]. Based on these pioneering studies, the model has been further improved in the last two decades, particularly by Wu and Yu [62] and Xu and Yu [63], and, more recently, a comprehensive study on this approach was proposed by Xiao et al. [64]. The model can be applied if the pore size distribution exhibits a symmetry over scales of magnitude, namely the increase in pore dimensions follows a defined scaling law with respect to the pore number, i.e., a fractal scaling law. In particular, the necessary precondition to apply the model is that $N\left(D\geq D_{MIN}\right)={\left(D_{MAX}/D_{MIN}\right)}^{d_{f}}$, where *N* is the number of pores whose diameters *D* are greater than or equal to *D_MIN_*, and *d_f_* is the fractal dimension (determined as a function of the porosity of the porous medium). The number of pores *N*, with diameters between *D_MIN_* and *D_MAX_*, obeying this condition, can be considered distributed according to the fractal scaling law with fractal dimension *d_f_* [65]. According to Xiao et al. [64], the fractal model can also be applied for porous materials with converging/diverging capillaries (i.e., pore narrowing) and with different surface roughness. This analytical fractal-based model allows estimating the permeability from the pore geometry and pore morphology distribution within porous materials, providing results compatible with experimental evidences.

## 5. Discussion

**Figure 7:**
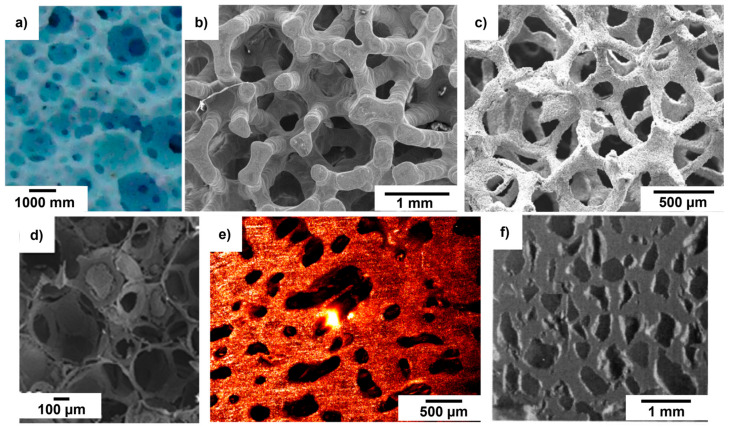
Examples of scaffold architectures for which the permeability has been assessed: (**a**) original image of HA cylindrical sample tested with pump-based method (reproduced from [45]); (**b**) SEM image of HA cylindrical scaffold used in the acoustic approach (reproduced from [50]); (**c**) 45S5 Bioglass^®^ scaffold used in the pump-based method (reproduced from [41]); (**d**) SEM image of 47.5B-based scaffolds tested with the acoustic approach (reproduced from [39]); (**e**) confocal microscopy image of calcium carbonate scaffold tested by acoustic approach (reproduced from [32]); (**f**) optical picture of macroporous biphasic calcium phosphate scaffolds used in the gravity-based approach (reproduced from [13]).

In the search for a global parameter describing the architecture and mass transport properties of porous biomaterials, permeability indeed emerges as a highly promising candidate. Experimental assessment of permeability often involves the use of distilled water as a flowing medium. If the possible damage of porous materials during the execution of the test is a serious concern owing to the risk of dissolution (e.g., in the case of bioactive glasses) or the weakness of the solid skeleton (e.g., in the case of ultra-porous bioceramic scaffolds), computational methods have also been developed that do not need any manipulation of the sample or contact with the liquid; however, micro-tomographic equipment is necessary to analyze and reconstruct the porous structure of the material to be used as the input to the fluid simulation software. Alternatively, an acoustic method has been developed that uses air and low-frequency pressure waves, proving to be highly reliable and repeatable. It is worth highlighting that the knowledge of intrinsic permeability combined with some pore characteristics (e.g., average pore size and pore sphericity, which can be assessed by scanning electron microscopy or even better by micro-computed tomography) is also useful to determine the complete set of microstructural properties of the scaffolds, including effective porosity and tortuosity, which can help materials scientists and bioengineers in the stage of scaffold design and optimization. In general, as displayed in Figure 7, a wide range of scaffold architectures can be investigated with regard to permeability and related microstructural parameters.

It cannot be ignored, however, that there is a lack of standardization in permeability measurement and testing procedure for scaffolds, which make it very difficult and partly unreliable to compare the results obtained in different laboratories (see also Table 1). At present, the only available and relevant standard was first published in 2014 (and revised in 2022); it describes a pump-based methodology to determine Darcian permeability [43]. However, this procedure requires the use of three transducers in the measurement system to assess upstream and downstream pressures and flow rate, and is not advisable for highly brittle or resorbable bioceramic scaffolds, as explained above. The acoustic method, other than requiring only a single transducer for the measurement (instead of three), allows overcoming these limitations, but, to date, it is still a non-standard approach (even if it can be partially linked to ISO Standard 9053-2:2020 procedures [66]). Despite this, measurement results obtained by the acoustic permeameter are highly compatible with data obtained by an ASTM-compliant method and really more precise, as widely discussed in [27]. In that comparison, two 3D-printed poly (lactic acid) (PLA) scaffolds (with a predefined geometry) and two commercial scaffolds based on bovine-derived mineral matrices were tested as “reference structured materials” by using both methods. Moreover, the detailed description of the comparison criteria can be considered a protocol draft supporting proficiency tests in other laboratories.

On the other hand, evidence of extensive comparisons between different methods, or ASTM-compliant methods, for bioceramic scaffolds is still lacking, along with proficiency tests. In this case, the possibility of exploiting newly developed “reference structured materials” could be very useful. In this regard, the reference range to use for making the comparison between permeability values is still a matter of debate. Data taken from natural bone are typically considered, but they are quite variable depending on harvesting site, sex and age. In recent years, the National Institute of Standards and Technology (NIST), in collaboration with the ASTM, has been actively working to identify and propose reference scaffolds for standardization in tissue engineering applications. The effort made by the NIST confirms the interest in the field, but, at present, such standards are still under development.

Recent scaffold fabrication technologies based on additive manufacturing, such as vat photopolymerization, could be implemented to fabricate highly accurate “reference structured materials” with reproducible and controlled geometry and internal architecture. Such materials would easily support comparisons between different measurement techniques in different laboratories, being handy, affordable, stable over time and structurally robust.

Comparisons and proficiency tests developed according to agreed protocols and procedures can be planned to provide evidence of measurement compatibility, also within involved standardization ASTM or ISO committees, collecting data from several laboratories worldwide. These evidences are expected to greatly improve the reliability of measurement results of the scaffold properties, which is a point of utmost importance being related to human health and safety.

Some authors tried formulating a dimensionless framework in which a sintered system can be compared with regard to permeability [67]; this approach also deserves to be considered and applied to bioceramic scaffolds.

## 6. Conclusions

The major conclusions from this review article and the relevant challenges ahead can be summarized as follows:

- Permeability can be used as a “global parameter” to describe the 3D porous architecture of scaffolds because it depends on a set of microstructural parameters including effective porosity, pore size distribution, pore tortuosity, etc.;
- There are three main classes of experimental techniques suitable to assess the permeability of bioceramic scaffolds, i.e., pump-based methods, gravity-based method and acoustic method, along with various computational approaches;
- Numerical methods can be used without any particular limitation, provided that an accurate 3D reconstruction of the porous biomaterial is available (e.g., using micro-tomographic imaging) and computational errors are kept in an acceptable range;
- To date, there is only one standardized procedure (published by ASTM) for assessing the permeability of porous scaffolds that refers to a pump-based method;
- The approach proposed by ASTM cannot be definitely claimed as superior to the other experimental techniques; on the contrary, the recently developed acoustic method seems to be preferable for highly fragile and resorbable scaffolds as it does not use any liquid medium that could damage or dissolve the material;
- In the authors’ opinion, the acoustic method and computational approaches are the most versatile strategies and can be potentially applied to any kind of ceramic material without particular constraints (see also the previous point), not limited to biomedical applications;
- The development of a “reference structured scaffold” for reliable comparative purposes is still to be finalized and would deserve to be discussed in the relevant regulatory bodies;
- The knowledge of permeability and major characteristics of pores (e.g., average pore assize and sphericity) allows determining the full set of microstructural parameters of the scaffolds, such as effective porosity and tortuosity, which are key for scaffold design;
- Simulation studies of the permeability at the bone/scaffold interface deserve to be carried out in future research.

## Figures and Tables

**Table 1:** Summary of the main studies dealing with permeability assessment discussed in this work.

Scaffold Material	Architecture and Size ^a^	Method for Permeability Measurement	Permeability (m^2^)	Reference
Hydroxyapatite	Foam-like cylinders (Φ = 30 mm, H = 20 mm)	Pump-based	(0.4–3.24) × 10^−9^	[45]
	Foam-like cylinders (Φ = 5 mm, H = 10 mm)	Acoustic	(1.25 ± 0.49) × 10^−9^	[50]
45S5 Bioglass^®^	Foam-like cuboids (L = 8 mm)	Pump-based	(1.96 ± 0.11) × 10^−9^	[41]
70S30C sol-gel bioactive glass	Foam-like cuboids	CFD	(580–767) × 10^−12^	[59]
Bioactive glass 47.5B	Trabecular-like cylinders (Φ = 8 mm, H = 7 mm)	Acoustic	(2.49–2.81) × 10^−10^	[39]
ProOsteon 500 HA (Interpore, Irvine, CA, USA)	Semi-regular trabecular structure (Φ = 6.5 mm, H = 10.5 mm)	Pump-based	(0.26 × 10^−9^ ± 2%)	[44]
Calcium carbonate	Low-porosity cylindrical scaffold (Φ = 15 mm, H = 30 mm)	Acoustic	(3.12 ± 0.11) × 10^−11^	[32]
Biphasic calcium phosphate	Macroporous scaffold with channel-shaped pores (L = 5 mm)	Gravity-based	(0.01–0.35) × 10^−9^	[13]
Sponceram^®^ (Zellwerk, Oberkraemer, Germany)	Porous scaffold disc	CFD	(17.9 ± 4.09) × 10^−9^	[58]

^a^ Dimensions of the scaffolds such as diameter (Φ), height (H) and length (L) are reported if available.

## Data Availability

This paper is a review; thus, original data can be found in the references cited in the manuscript.
